# *N* ^6^-Hydroperoxymethyladenosine: a new intermediate of chemical oxidation of *N*^6^-methyladenosine mediated by bicarbonate-activated hydrogen peroxide†
†Electronic supplementary information (ESI) available: Methods, figures and spectra are included. See DOI: 10.1039/c5sc00484e



**DOI:** 10.1039/c5sc00484e

**Published:** 2015-03-11

**Authors:** Jinjun Wu, Heng Xiao, Tianlu Wang, Tingting Hong, Boshi Fu, Dongsheng Bai, Zhiyong He, Shuang Peng, Xiwen Xing, Jianlin Hu, Pu Guo, Xiang Zhou

**Affiliations:** a College of Chemistry and Molecular Sciences , Institute of Advanced Studies , Wuhan University , Wuhan , Hubei 430072 , P. R. China . Email: xzhou@whu.edu.cn ; Fax: +86-27-68756663 ; Tel: +86-27-68756663; b State Key Laboratory of Natural and Biomimetic Drugs , Peking University , Beijing , 100191 , P. R. China

## Abstract

*N* ^6^-Methyladenosine (m^6^A) represents a relatively abundant modification in eukaryotic RNA. Because m^6^A has similar properties to adenosine and a low reactivity, limited research has been focused on this nucleoside. In this study, we revealed an important intermediate in the oxidation of m^6^A through the bicarbonate-activated peroxide system. Over the course of oxidation, we found a new mechanism in which *N*^6^-hydroxymethyladenosine (hm^6^A), *N*^6^-formyladenosine (f^6^A) and *N*^6^-hydroperoxymethyladenosine (oxm^6^A) were intermediate products, and adenosine was the final product. In this study, oxm^6^A was isolated using HPLC and characterized by mass spectrometry, NMR and diphenyl-1-pyrenylphosphine (DPPP) fluorescence detection. This study provides a new modified nucleoside and demonstrates oxidative demethylation of m^6^A by reactive oxygen species at the nucleobase level and in RNA strands.

## Abstract 2

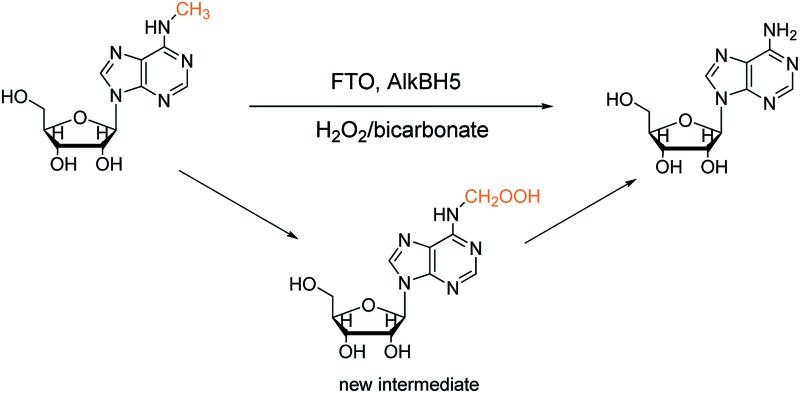
A new route is found in the chemical oxidation of *N*^6^-methyladenosine using a H_2_O_2_/bicarbonate system through the *N*^6^-hydroperoxymethyladenosine (oxm^6^A) intermediate.

## 

*N* ^6^-Methyladenosine represents the most abundant modification in the mRNA of higher eukaryotes, present at a frequency of approximately three sites on each mRNA.^1^ m^6^A is also present on tRNA, rRNA and lnRNA.^2^ This modification plays an important role in the regulation of gene expression.^3^ Since its discovery last century,^4^ m^6^A has been the object of relatively few studies. Recently, fat mass- and obesity-associated proteins (FTO)^5^ and AlkBH5 ^6^ were found to be m^6^A demethylases, indicating a novel regulatory mechanism in mammalian cells. Two new modifications, *N*^6^-hydroxymethyladenosine (hm^6^A) and *N*^6^-formyladenosine (f^6^A), have been found to participate in the FTO-mediated demethylation process, which may influence RNA–protein interactions and regulate gene expression.^7^ In addition, transcriptome-wide profiling of m^6^A in mRNA and lnRNA has revealed new insights into the role of RNA modification.^8^ These developments have renewed interest in the investigation of this particular, distinctive modification. Therefore, we aspire to use a chemical method to differentiate m^6^A from A.

Hydrogen peroxide is a widely used oxidant with a high content of active oxygen,^9^ but its relatively slow oxidizing rate limits its usage. Bicarbonate is present in cells and serum at high concentrations, ranging from 14.7–25 mM,^10^ and plays an important role in biological oxidation.^11^ H_2_O_2_ and NH_4_HCO_3_ are environmentally friendly reagents; H_2_O_2_ produces only water as a by-product, and NH_4_HCO_3_ easily decomposes to NH_3_, CO_2_, and H_2_O. The reaction conditions are mild at natural pH values.

Owing to its high reactivity towards secondary amines, we considered whether the oxidant could react with m^6^A. Surprisingly, instead of producing *N*-oxides, demethylated adenosine was produced, and the presence of several intermediates in the reaction system suggested a potential mechanism in the chemical reaction (Scheme 1). These results suggest that H_2_O_2_/bicarbonate can act as a reactive oxygen species (ROS) for demethylation. In this study, we determine a key intermediate in the demethylation process, and we investigate the underlying mechanism.

**Scheme 1:**
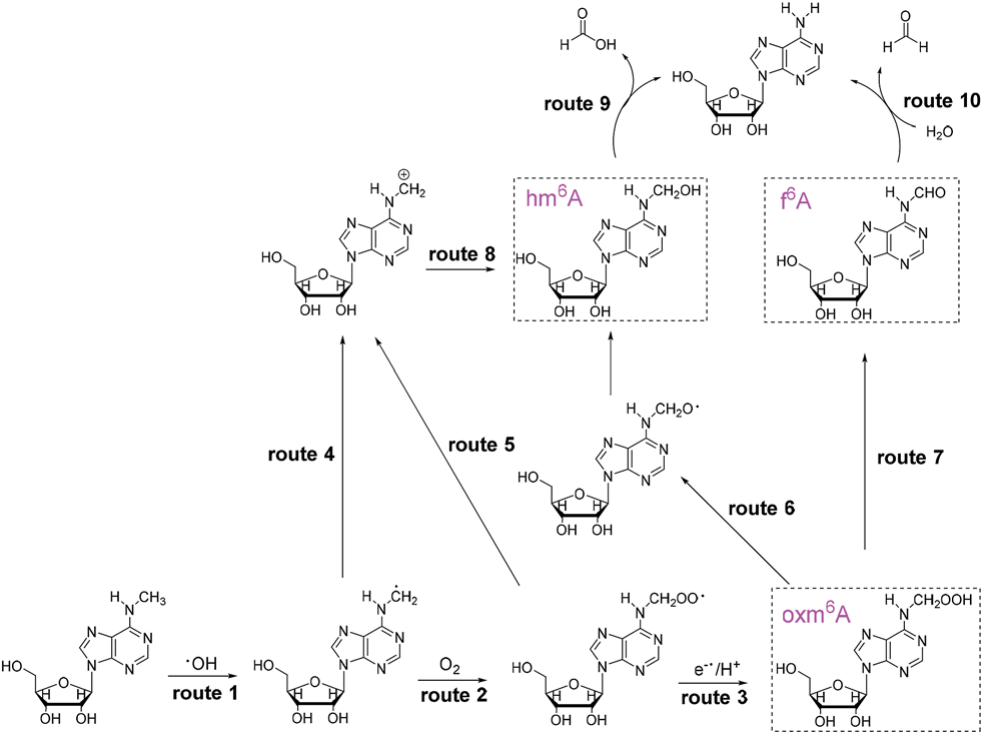
Proposed mechanism of the demethylation process, and structures of oxidation products.

To investigate the demethylation process, we used high-performance liquid chromatography (HPLC) to monitor the reaction (UV detector at 260 nm). When a 2 mM aliquot of m^6^A was treated with 200 mM H_2_O_2_ and 1 M NH_4_HCO_3_ at 37 °C for one hour, four products were formed: A, hm^6^A, oxm^6^A and f^6^A (Fig. 1). The LC-MS data showed masses corresponding to A (267.9), hm^6^A (297.8), oxm^6^A (313.8) and f^6^A (295.9), successively in the positive-ion mode (Fig. S1†). Product A was further characterized by ^1^H and ^13^C NMR (see ESI†). To confirm the occurrence of hm^6^A and f^6^A, these compounds were synthesized according to reported procedures.^7^ An equilibrium reaction between adenosine and formaldehyde produced hm^6^A (Scheme 1, Route 9). Further HPLC analysis indicated that the synthesized hm^6^A and f^6^A have the same retention times as the reported hm^6^A and f^6^A, respectively (Fig. 1b–d). We found that hm^6^A and f^6^A were unstable and could decompose to A (adenosine) during HPLC analysis (Fig. 1c and d). *N*^6^-Hydroperoxymethyladenosine (oxm^6^A) was found to be a new intermediate, in addition to hm^6^A and f^6^A, during the demethylation of m^6^A (Fig. 1b). When we incubated the m^6^A with bicarbonate or H_2_O_2_ alone, no reaction was observed (Fig. S2 and S3†).

**Fig. 1:**
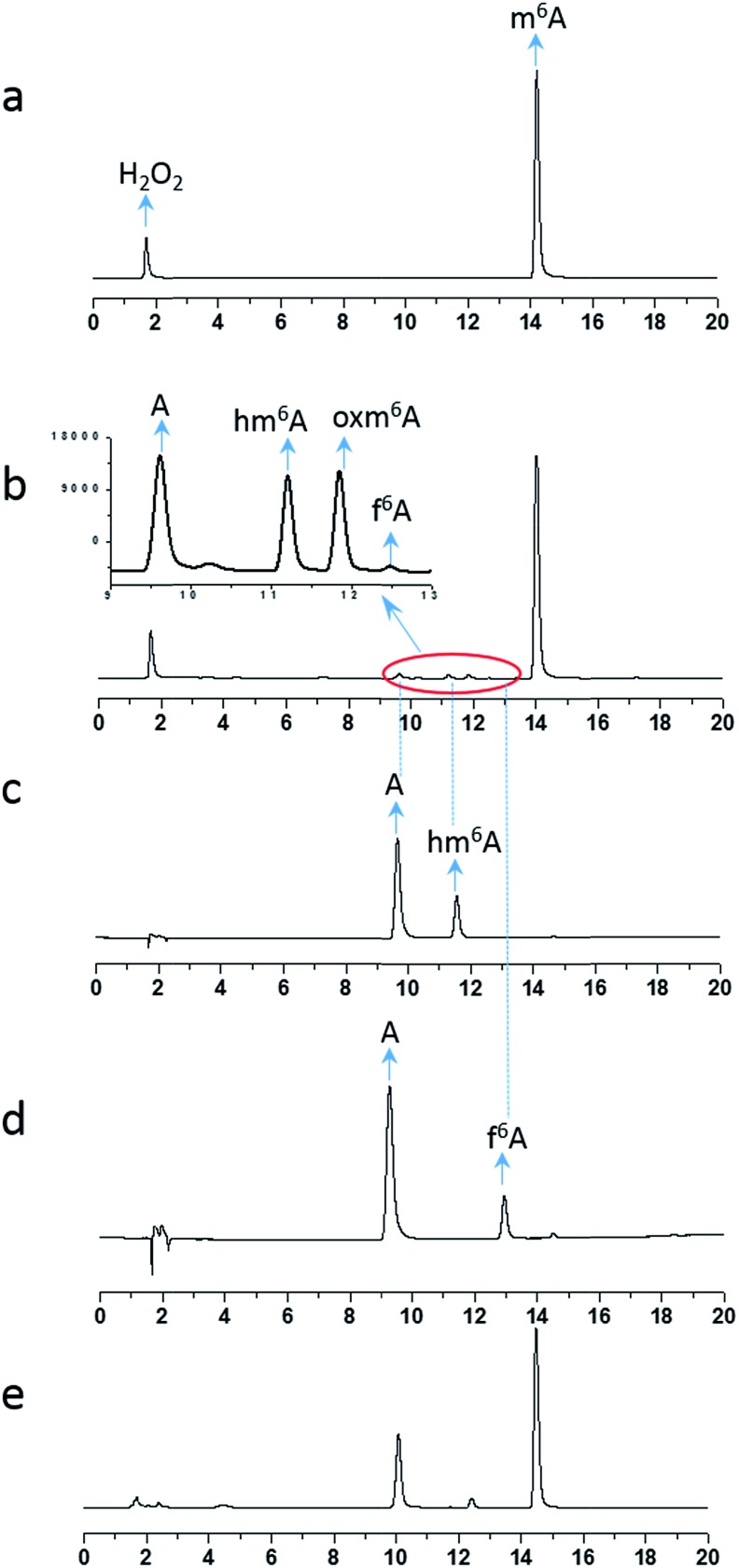
HPLC chromatograph of 2 mM m^6^A incubated with 200 mM H_2_O_2_ and 1 M NH_4_HCO_3_ at 37 °C for 0 h (a), 1 h (b) and 24 h (e). As shown in the HPLC profiles, when the reaction proceeded for 1 h, one major product (A) was produced, accompanied by three intermediates (hm^6^A, f^6^A, oxm^6^A). The synthesized hm^6^A (c) and f^6^A (d) standards have the same retention time as two of the new peaks in the reaction mixture. Because hm^6^A and f^6^A are unstable, they can coexist with A during HPLC analysis.

Diphenyl-1-pyrenylphosphine (DPPP), as a fluorescent reagent, can be used for hydroperoxide determinations.^12^ When we incubated the intermediate with DPPP in the presence of butylated hydroxytoluene (BHT) at 37 °C for 1 h, the fluorescence increased, indicating the formation of a hydroperoxide intermediate (Fig. 2). Further characterization of oxm^6^A was achieved using high-resolution mass spectrometry, ^1^H NMR, ^13^C NMR and TOCSY (ESI, Fig. 3, S4 and S5†), with the corresponding chemical structures shown in Scheme 1. To confirm the chemical shifts of the protons in N–H and OO–H, ^1^H NMR was performed in DMSO-d_6_ and in D_2_O. In the DMSO-d_6_ solution, the chemical shifts of the protons were 8.61 ppm (–N–H) and 11.71 ppm (–OOH) (Table 1). To confirm our hypothesis, we changed the solution to D_2_O, where deuterons can be incorporated at the N–H and O–H positions because of hydrogen–deuterium (H/D) exchange behavior. As we expected, these two protons disappeared in the D_2_O solution (Fig. 3). We then used total correlation spectroscopy (TOCSY) to show the H–H correlation; the TOCSY spectrum was acquired using a 600 MHz Bruker Avance II spectrometer equipped with a 5 mm triple resonance cryoprobe. The pulse sequence was DIPSI2ETGP. The relaxation delay was 1 s, with 8 acquisitions per increment, and a spectral width of 8 × 8 ppm and time domain of 2*k* × 176 were used. In the spectrum, the NH proton had a cross peak with CH_2_ at *δ* (8.66, 5.26 ppm), further confirming the oxm^6^A structure. When we analysed the reaction mixture using LC-MS, we detected a relatively small mass signal of 311.8; this finding may indicate the generation of another intermediate, *N*^6^-carboxyladenosine, in a relatively low yield (Fig. S1c†). Meanwhile, our control experiments indicated that adenosine, uridine, cytidine and guanosine were stable in the H_2_O_2_/bicarbonate solution at concentrations of 200 mM H_2_O_2_ and 1 M NH_4_HCO_3_ (Fig. S6†) after one hour.

**Fig. 2:**
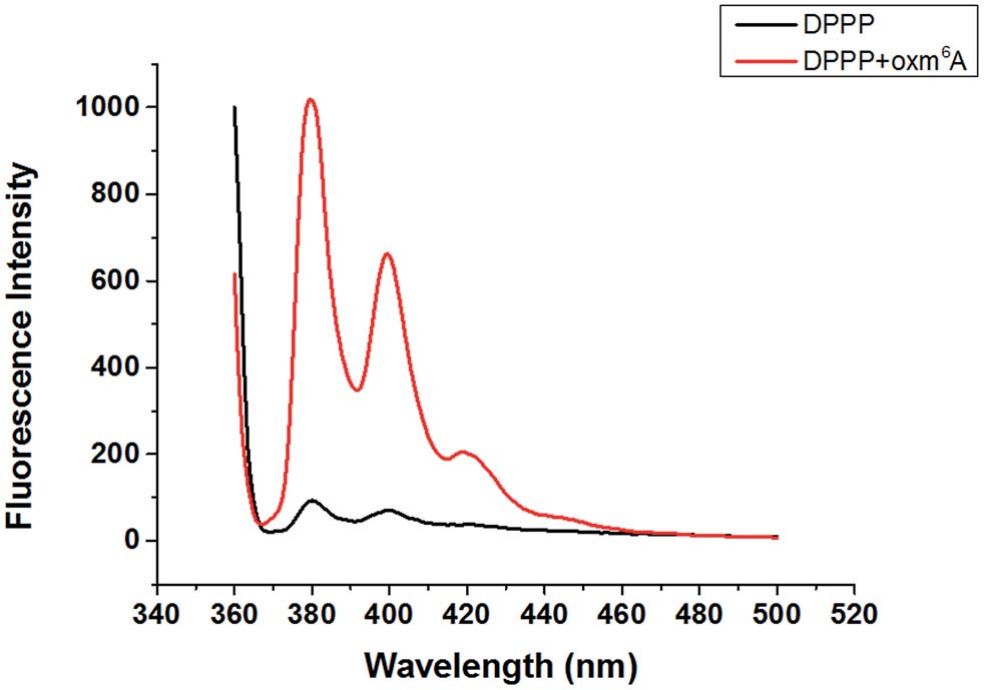
Fluorescence emission spectra (*λ*_ex_ = 352 nm) of DPPP in the presence of (a) and in the absence of oxm^6^A (b) after incubation with BHT at 37 °C for 60 min.

**Fig. 3:**
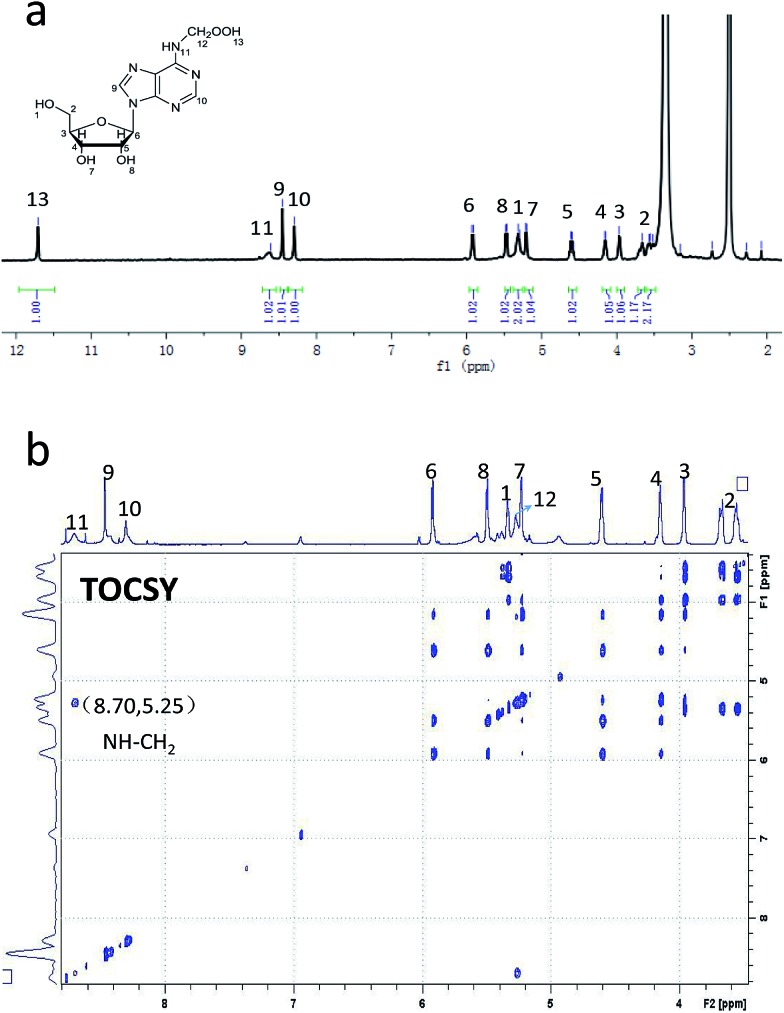
^1^H NMR spectrum (a) and TOCSY spectrum (b) of oxm^6^A in DMSO-d_6_.

**Table 1:** ^1^H chemical shifts (*δ*, ppm) of oxm^6^A in DMSO-d_6_ at room temperature

Number	Chemical shift (*δ*, ppm)	Number	Chemical shift (*δ*, ppm)	Number	Chemical shift (*δ*, ppm)
1	5.33	6	5.91	11	8.66
2	3.68	7	5.23	12	5.26
3	3.96	8	5.50	13	11.72
4	4.14	9	8.46		
5	4.61	10	8.31		

Because our goal was to fully investigate the mechanism of m^6^A demethylation, we extended the reaction time to 24 hours. After 24 hours, we found that only A (primary product) and a small amount of oxm^6^A were present (Fig. 1e), whereas hm^6^A and f^6^A disappeared. This result suggested that hm^6^A and f^6^A were converted into A (Scheme 1, Routes 9 and 10).

To investigate the behaviour of oxm^6^A, it was separated from the reaction mixture, incubated in HEPES buffer (50 mM, pH 7.4) at 37 °C and then analysed by HPLC every 2 h. We found that the amount of A increased at the expense of oxm^6^A (Fig. S7 in the ESI†), and it had a half-time of approximately 8.5 h (Fig. S8†), which was more stable than hm^6^A and f^6^A (approximately 3 h).

In the H_2_O_2_/NH_4_HCO_3_ system, the hydroxyl radical was trapped by 5,5-dimethyl-1-pyrroline-*N*-oxide (DMPO) to give a signal using Electron Paramagnetic Resonance (EPR) (Fig. S9†). In the reaction system, the addition of DMSO, a hydroxyl radical scavenger, dramatically decreased the chemical demethylation level of m^6^A (Fig. S10†). We speculate that the reaction underwent a hydroxyl radical mechanism. A hydroxyl radical abstracted a hydrogen atom from a methyl group to yield a carbon radical, which could then bind with O_2_ to form oxm^6^A (Scheme 1, Routes 2 and 3) or bind with ˙OH to form hm^6^A (Scheme 1, Routes 4 and 8), parallel to the decomposition mechanism for 5′-hydroperoxymethyluracil and 5′-hydroperoxymethylcytosine, as proposed by Richard Wagner's group.^13^ To confirm the possibility of the ˙OH radical mechanism, we used Fenton-type reagents to react with m^6^A. The formation of hm^6^A, oxm^6^A, f^6^A and A was also observed using LC-MS analysis, confirming the reaction mechanism (Fig. S18†). Under identical experimental conditions but with the addition of a small amount of (NH_4_)_2_Fe(SO_4_)_2_ in the H_2_O_2_/NH_4_HCO_3_ reaction mixture, the reaction rate markedly increased (Fig. S11†). As the reaction is based on the hydroxyl radical mechanism, and Fe^2+^ as well as Cu^2+^ have great influences on the reaction, we therefore used Inductively Coupled Plasma Optical Emission Spectroscopy (ICP-OES) to investigate the presence of iron(ii) and copper(ii) in the H_2_O_2_/bicarbonate reaction system. No signals were observed, and both the concentration of Fe^2+^ and Cu^2+^ were lower than 10 ng mL^–1^, indicating the reaction could proceed with just a bicarbonate-activated peroxide system (the optimized operating conditions are shown in Table S1†). In the demethylation process, two pathways are shown. A hydroxyl radical attacks the methyl radical to form hm^6^A (Scheme 1, Routes 4 and 8) and O_2_ attacks the methyl radical to form oxm^6^A (Scheme 1, Routes 2 and 3). The oxm^6^A and its peroxide radical can decompose to hm^6^A (Scheme 1, Routes 5, 8 and 6) and f^6^A (Scheme 1, Route 7), and we propose that the new route in the demethylation process would improve the efficiency of the demethylation reaction compared to just attacking the methyl radical by a hydroxyl radical.

Next, because m^6^A is preferentially present in the consensus sequence RRm^6^ACH (R is A/G and H is A/C/U),^14^ to examine whether the reaction occurs in RNA oligos, we prepared a 9-mer oligoribonucleotide (5′-CUGGm^6^ACUGG-3′) containing one m^6^A site and treated it with 10 mM H_2_O_2_ and 100 mM bicarbonate at 37 °C for 48 h. Because RNA may decompose in a high concentration of H_2_O_2_, we decreased the concentration of H_2_O_2_ and NH_4_HCO_3_. After the reaction, the oligo RNA was analysed using MALDI-TOF mass spectrometry as shown in Fig. S12.† We found a m^6^A –14 Da peak, representing a demethylation product, as well as a +14 Da peak and a +17 Da peak, which may correspond to *N*^6^-formyladenosine and *N*^6^-hydroxymethyladenosine intermediates in the demethylation pathway, respectively. At natural pH levels (pH 7.4), hm^6^A, oxm^6^A and f^6^A were relatively stable, but an alkaline phosphate digestion may accelerate their decomposition. Therefore, to verify the presence of hm^6^A, oxm^6^A and f^6^A in the oligo RNA after the reaction, we used RNase T1 followed by nuclease P1 to digest the oligo RNA,^7^ then analysed the reaction using LC-MS. In this analysis, RNase T1 can selectively digest the phosphodiester bond after G. We successfully detected the formation of A, hm^6^A, oxm^6^A, and f^6^A in the digested nucleoside, similar to our proposed mechanism for a single nucleoside (Fig. S13†).

To explore the reaction kinetics of the oxidation, two micrograms of oligo RNA were incubated with 100 μM H_2_O_2_ and 300 μM NH_4_HCO_3_ at 37 °C for 30 h in six parallel experiments, followed by digestion with nuclease P1 and alkaline phosphate. The amount of A generated from m^6^A was quantified using LC-MS every 3 hours (the calibration curve is shown in the ESI, Fig. S14†). As depicted in Fig. S15,† the A content exhibited a strong linear relationship with reaction time over a period of 30 hours. After adding Fe^2+^ to the H_2_O_2_/NH_4_HCO_3_ mixture and incubating it with oligo RNA, HPLC analysis of the enzymatically digested nucleosides in RNA showed the presence of demethylated adenosine with a decreased level of m^6^A after oxidation for 1 h (Fig. S16†).

Although FTO-mediated oxidation of m^6^A may decrease the level of m^6^A *in vitro*, no *in vitro* experiments have been reported in which a chemical reagent was used to demethylate m^6^A. We explored whether m^6^A in genomic RNA is a substrate of H_2_O_2_/NH_4_HCO_3_*in vitro*. Total RNA was extracted from Hela cells using the TRIzol reagent (Invitrogen) according to the manufacturer's protocol. Four micrograms of genomic RNA was incubated with 100 μM H_2_O_2_ and 1 mM NH_4_HCO_3_ at 37 °C for 12 h. After digestion with nuclease P1 and alkaline phosphatase, the solution was analysed by LC-MS. The results showed a decrease in the m^6^A/A ratio by 10% in the genomic RNA (Fig. S17†), indicating that the reagents demethylated m^6^A *in vitro*.

## Conclusions

In conclusion, we reported a new chemical method for the oxidative demethylation of m^6^A and determined an important intermediate in the reaction system. Three intermediates, *N*^6^-hydroxymethyladenosine (hm^6^A), *N*^6^-formyladenosine (f^6^A), and *N*^6^-hydroperoxymethyladenosine (oxm^6^A), were characterized, and the mechanism underlying the decomposition was illustrated. We also determined that the reaction could occur in oligo RNA and genomic RNA *in vitro*. H_2_O_2_ is a reactive oxygen species that is endogenously produced during normal metabolism^15^ and immune responses,^16^ and a high concentration of bicarbonate is found in cells and serum. Thus, this route may occur *in vivo* and play a role in cells. ROS have been proven to directly react with genomic DNA in a chemical reaction.^17^ Recently, reports have shown that ROS can induce the oxidative conversion of 5mC to 5hmC in a TET dioxygenase-dependent manner,^18^ indicating ROS regulate the enzymatic catalytic reaction. We propose that the oxm^6^A was formed through direct oxidation by ROS *in vivo*, just like the nucleoside analogues formed in RNA induced by Fenton-type reagents.^17*b*^ Further study is in progress to study the presence and biological function of oxm^6^A *in vivo*. The discovery of the new intermediate oxm^6^A and the chemical route for the demethylation of m^6^A to A may offer new insight into the study of m^6^A.

## Supplementary Material

Supplementary information
