# Supplementary material for: N 6-Hydroperoxymethyladenosine: a new intermediate of chemical oxidation of N6-methyladenosine mediated by bicarbonate-activated hydrogen peroxide
Source: Chem Sci. 2015 Mar 11;6(5):3013–7. doi: 10.1039/c5sc00484e (PMC5657413; doi:10.1039/c5sc00484e)
Supplement: Supplementary file 1 [file SC-006-C5SC00484E-s001.pdf]

Electronic Supplementary Material (ESI) for Chemical Science.
This journal is © The Royal Society of Chemistry 2015

Supporting Information

for

N[6]-hydroperoxymethyladenosine: a new intermediate of chemical oxidation of *N*[6]-methyladenosine mediated by bicarbonate-activated hydrogen peroxide

Jinjun Wu,‡ Heng Xiao,‡ Tianlu Wang,‡ Tingting Hong, Boshi Fu, Dongsheng Bai, Zhiyong He, Shuang Peng, Xiwen Xing, Jianling Hu, Pu Guo, Xiang Zhou*

College of Chemistry and Molecular Sciences,

Institute of Advanced Studies,,

Wuhan University Wuhan, Hubei, 430072, (P. R. of China)

State Key Laboratory of Natural and Biomimetic Drugs, Peking University, Beijing, 100191, P. R. China.

E-mail: xzhou@whu.edu.cn

Methods

Materials.

All chemicals were of analytical reagent grade. Cytidine (C), adenosine (A), guanosine (G), uridine (U), ammonium bicarbonate (NH_4HCO_3), nuclease P1 and nuclease T1 were purchased from Sigma. N^6-methyladenosine (m^6A) was purchased from Shanghai Civi Chemical Technology Co., Ltd. Hydrogen peroxide, sodium chloride, zinc chloride and formic acid were obtained from Sinopharm Chemical Reagent. Shrimp alkaline phosphate (SAP) was from New England Biolabs. The oligo RNA was kindly offered by Professor Chuan He's group at University of Chicago. ^1H and ^{13}C NMR spectra were recorded on Varian Mercury 300 spectrometers, respectively. TOCSY recorded on Varian Mercury 600 spectrometers. EPR was recorded on Bruker BioSpin GmbH, HRMS was recorded on Thermo Fisher LTO Orbitrap XL. HPLC data was collected with Lambo Model 2000. LC-MS data was recorded on Agilent LC/MSD. MALDI-TOF MS spectra were collected with an Axima-TOF_2 mass spectrometer (Shimadzu, Japan).

Demethylation of *N*$^{6-}$methyladenosine (m^6A) to form adenosine (A).

To obtain the ^1H and ^{13}C NMR spectra of the oxidation product, N^6-methyladenosine (m^6A, 281mg, 1mmol), was added to a stirred solution with 5.66mL 30%H_2O_2 (50mmol) and 1.975g NH_4HCO_3 (25mmol) in 25mL solution at room temperature, and stirred for 1h.[1] Then the reaction mixture was purified by column chromatography (SiO_2, EtOAc/methanol = 8:1 as eluents) to yield 110mg (39%) adenosine (A) as a white solid.[2] ^1H NMR (300 MHz, DMSO) δ(ppm): 8.36 (s, 1H), 8.14 (s, 1H), 7.39 (s, 2H), 5.88 (d, *J* = 5.7 Hz, 1H), 5.47 (d, *J* = 6.0 Hz, 2H), 5.21 (d, *J* = 4.2 Hz, 1H), 4.62 (m, 1H), 4.14 (m, 1H), 3.97 (m, 1H), 3.66 (m, 1H), 3.55 (m, 1H). ^{13}C NMR (75 MHz, DMSO) δ(ppm): 155.5, 151.8, 148.4, 139.3, 118.7, 87.2, 85.2, 72.8, 70.0, 61.0; LC-MS (m/z) for $C_{10}H_{13}N_5O_4$ $[M+H]^+$: 268.1 (calculated), 267.9 (found).

Synthesis of *N*^{6}-hydroxymethyladenosine (hm^6A) and *N*^{6}-formyladenosine (f^6A) standard.

The hm^6A and f^6A standard was synthesized according to the reported procedure.[3] The hm^6A was characterized using LC-MS. Characterization data of hm^6A: LC-MS (m/z) for $C_{11}H_{15}N_5O_5$ $[M+H]^+$: 298.1 (calculated), 297.8 (found). The f^6A was characterized by ^1H NMR and LC-MS. Characterization data of f^6A: ^1H NMR (300 MHz, DMSO) δ 11.34 (s, 1H), 9.92 (s, 1H), 8.74 (s, 1H), 8.59 (s, 1H), 6.00 (d, *J* = 5.5 Hz, 1H), 5.52 (s, 1H), 5.23 (s, 1H), 5.12 (s, 1H), 4.58 (t, *J* = 4.9 Hz, 1H), 4.15 (s, 1H), 3.96 (d, *J* = 3.5 Hz, 1H), 3.67 (d, *J* = 10.8 Hz, 1H), 3.53 (m, 1H). LC-MS (m/z) for $C_{11}H_{13}N_5O_5$ $[M+H]^+$: 296.1 (calculated), 295.8 (found).

Characterization of *N*^{6}- hydroperoxymethyladenosine (oxm^6A).

2mM m^6A was reacted with 200mM H_2O_2 and 1M NH_4HCO_3 at 37^oC for 1h, the oxm^6A was isolated and characterized by LC-MS, high-resolution mass spectra and both ^1H NMR , ^{13}C NMR and TOCSY. Characterization data of oxm^6A: ^1H NMR (300 MHz, DMSO) δ 11.71 (s, 1H), 8.61 (d, *J* = 9.0 Hz, 1H), 8.45 (s, 1H), 8.29 (s, 1H), 5.92 (d, *J* = 6.0 Hz, 1H), 5.47 (d, *J* = 6.0 Hz, 1H), 5.31 (t, *J* = 11.1 Hz, 2H), 5.21 (d, *J* = 4.5 Hz, 1H), 4.60 (dd, *J* = 5.4 Hz, 1H), 4.15 (d, *J* = 3.6 Hz, 1H), 3.97 (d, *J* =3.0Hz, 1H), 3.68 (m, 1H), 3.54 (m, 2H). ^1H NMR (300 MHz, D_2O) δ 8.36 (s, 1H), 8.34 (s, 1H), 6.08 (s, 1H), 5.46 (s, 2H), 4.43 (s, 1H), 4.28 (s, 1H), 3.87(d, *J* = 13.8 Hz, 2H). ^{13}C NMR (75 MHz, DMSO) δ(ppm): 155.0, 152.8, 150.4, 141.2, 120.6, 88.5, 86.5, 76.0, 74.3, 71.2, 62.3; LC-MS (m/z) for $C_{11}H_{15}N_5O_6$ $[M+H]^+$: 314.1 (calculated), 313.8 (found). High-resolution MS (m/z) for $C_{11}H_{15}N_5O_6$ $[M+H]^+$: 314.10951 (calculated), 314.10958 (found).

The reaction between m^6A and H_2O_2/bicarbonate analyzed by HPLC.

2mM m^6A was incubated with 200mM H_2O_2 and 1M NH_4HCO_3 at 37°C for 24h. Then the mixture was analyzed on a HPLC system equipped with an Agilent Eclipse XDB-C18 analysis column (150×4.6 mm) with mobile phase A (H_2O) and B (CH_3CN) with a flow rate of 1mL/min at room temperature. The separation was achieved with following gradient program: 15min 2%-12% B, 10min 12% B, and 10min 2% B. The detection wavelength was set as 260 nm.

MALDI-TOF-MS analysis of RNA oligo.

Two microgram oligo RNA was incubated with 10mM H_2O_2 and 100 mM NH_4HCO_3 at 37°C for 48h. The reaction solution was desalted by using ammonium-charged AG 50 W-X8 Cation Exchange Resins (Bio-Rad). MALDI-TOF MS spectra were collected in negative mode.

Analysis of hm^6A, fm^6A and oxm^6A in oligo RNA using LC-MS.

Two microgram oligo RNA was incubated with 10 mM H_2O_2 and 10 mM NH_4HCO_3 at 37°C for 1h, then the RNA was digested with nuclease T1 at 37°C for 15min in 50uL solution, followed by the nuclease P1 digestion for 30min. The solution was diluted five times, 50uL solution was analyzed by LC-MS equipped with a Hisep C18-T column (150mm×2.1 mm) with a flow rate of 0.2mL/min at 37°C. 0.01% formic acid in methanol (buffer A) and 0.01% formic acid in H_2O (buffer B) were applied as mobile phase. A gradient of 3min 5% B, 7min 5%-20% B, 6min 20% B, 5min 50% B and 3min 50%-5% B was used. The mass spectrometry detection was performed under single ion monitoring mode (SIM). LC-MS (m/z) for m^6A $[M + H]^+$: 282.1 (calculated), 281.9 (found), A $[M + H]^+$: 268.1 (calculated), 267.8 (found), hm^6A $[M+H]^+$: 298.1 (calculated), 297.8 (found), f^6A $[M + H]^+$: 296.1 (calculated), 295.8 (found), oxm^6A $[M+H]^+$: 314.1(calculated), 313.8 (found).

LC-MS analysis of m^6A demethylation in vitro.

Total RNA was extracted from Hela cells using TRIzol reagent (Invitrogen) according to the manufacture's protocol. The RNA concentration was measured by NanoDrop. Four microgram of genome RNA was incubated with 100 μM H_2O_2 and 1mM NH_4HCO_3 at 37°C for 12 hours. Nuclease P1 (1U) was added to the 50uL solution containing 25mM NaCl and 2.5mM $ZnCl_2$ for 1h and followed by alkaline phosphatase digestion (1U) at 37°C for 1h. Dilute the solution five times, 50uL solution was analyzed by LC-MS.

Determination of oxm^6A with Fluorometric Reagent Diphenyl-1-pyrenylphosphine.

The oxm^6A is dissolved in a mixture of methanol and water (1:1), and 100uL butylated hydroxytoluene (BHT) (1mg/10mL in a methanol-chloroform (1:1) and 100uL DPPP solution (1mg/10mL in a methanol-chloroform (1:1) are added. Then the mixture was reacted in a water bath at 37°C for 60min in dark. The solution was diluted 10 times and measured the fluorescence intensity at 380nm.

a

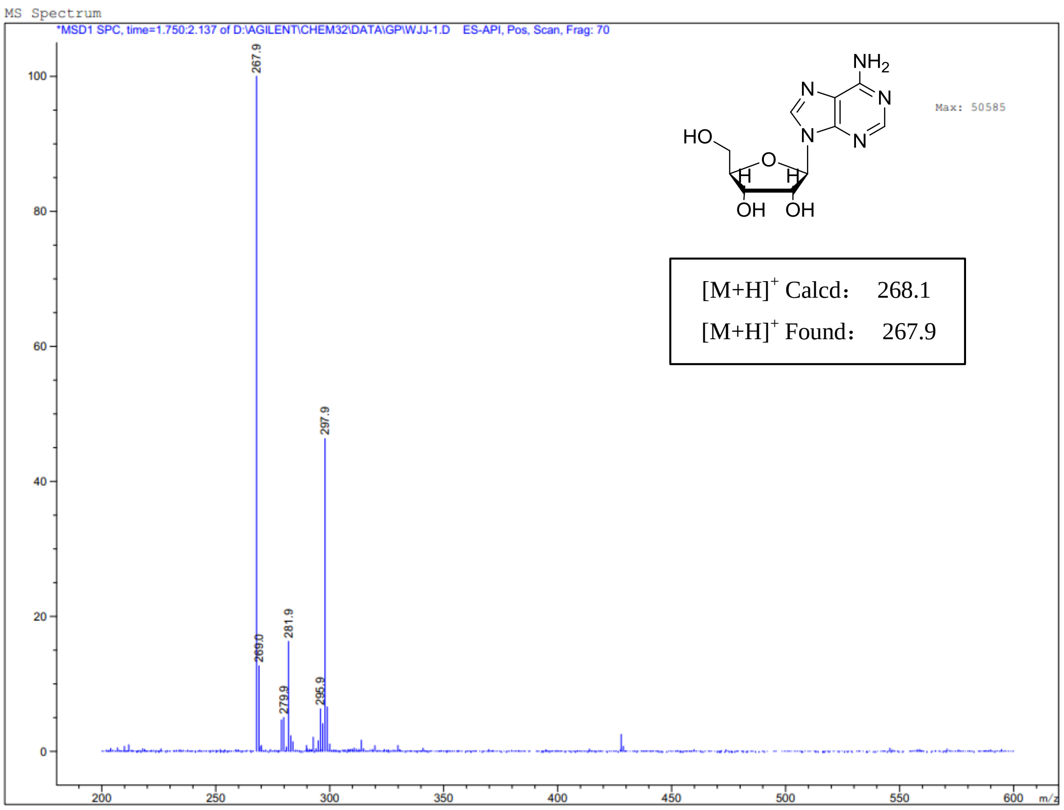
MS Spectrum
*MSD1 SPC, time=1.750:2.137 of D:\AGILENT\CHEM32\DATA\GP\WJJ-1.D ES-API, Pos, Scan, Frag: 70
Max: 50585
[M+H]+ Calcd: 268.1
[M+H]+ Found: 267.9
267.9
297.9
281.9
269.0
279.9
295.9
m/z

b

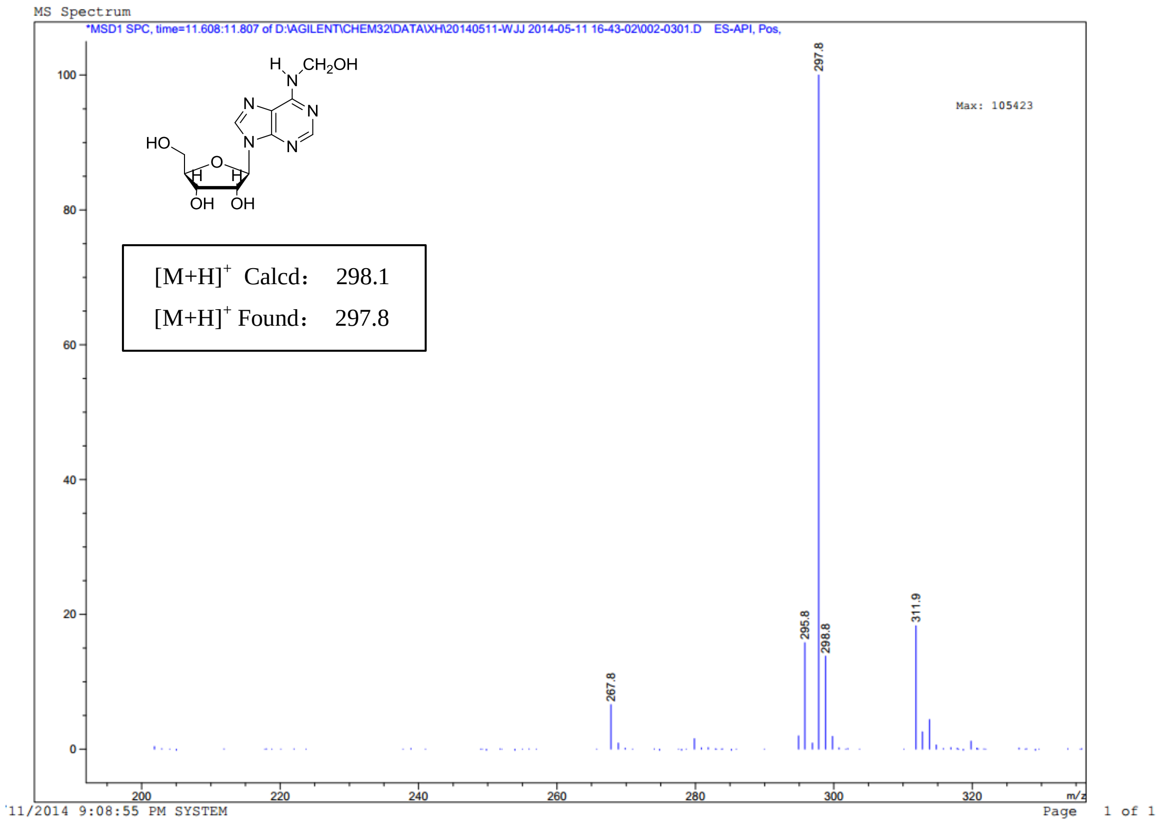
MS Spectrum
*MSD1 SPC, time=11.608:11.807 of D:\AGILENT\CHEM32\DATA\XH\20140511-WJJ 2014-05-11 16-43-02\002-0301.D ES-API, Pos,
Max: 105423
[M+H]+ Calcd: 298.1
[M+H]+ Found: 297.8
297.8
311.9
295.8
298.8
267.8
m/z
'11/2014 9:08:55 PM SYSTEM
Page 1 of 1

c

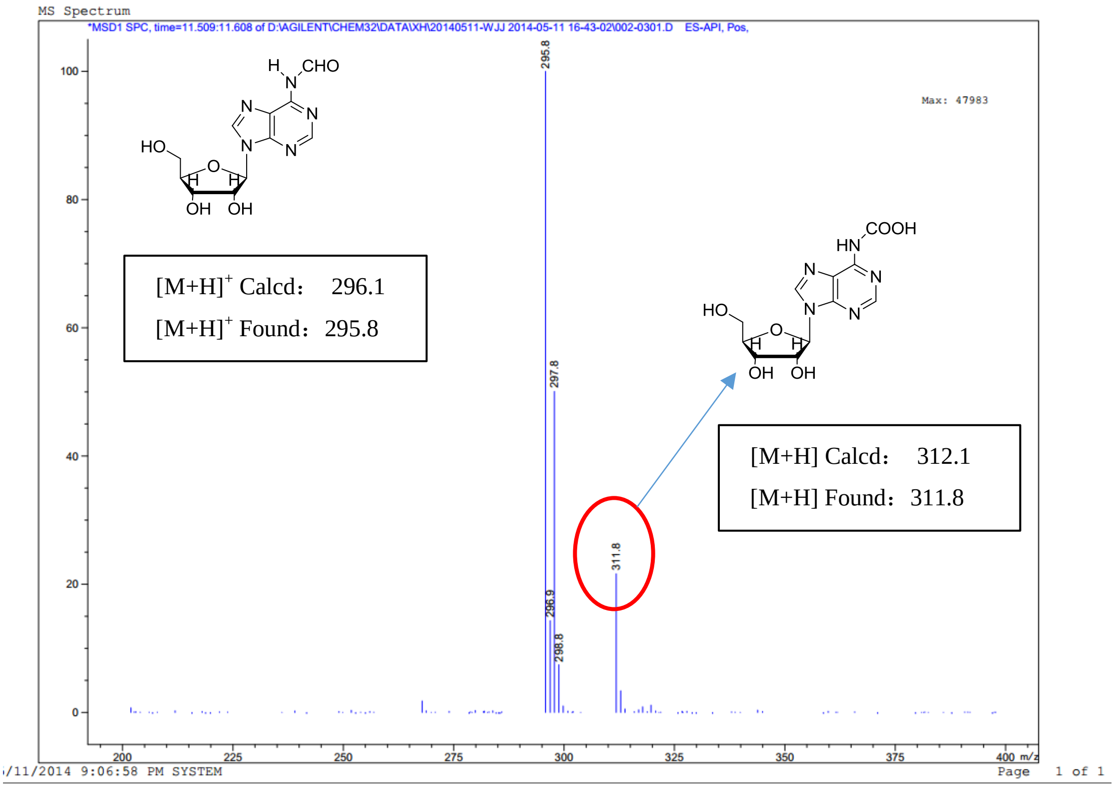

d

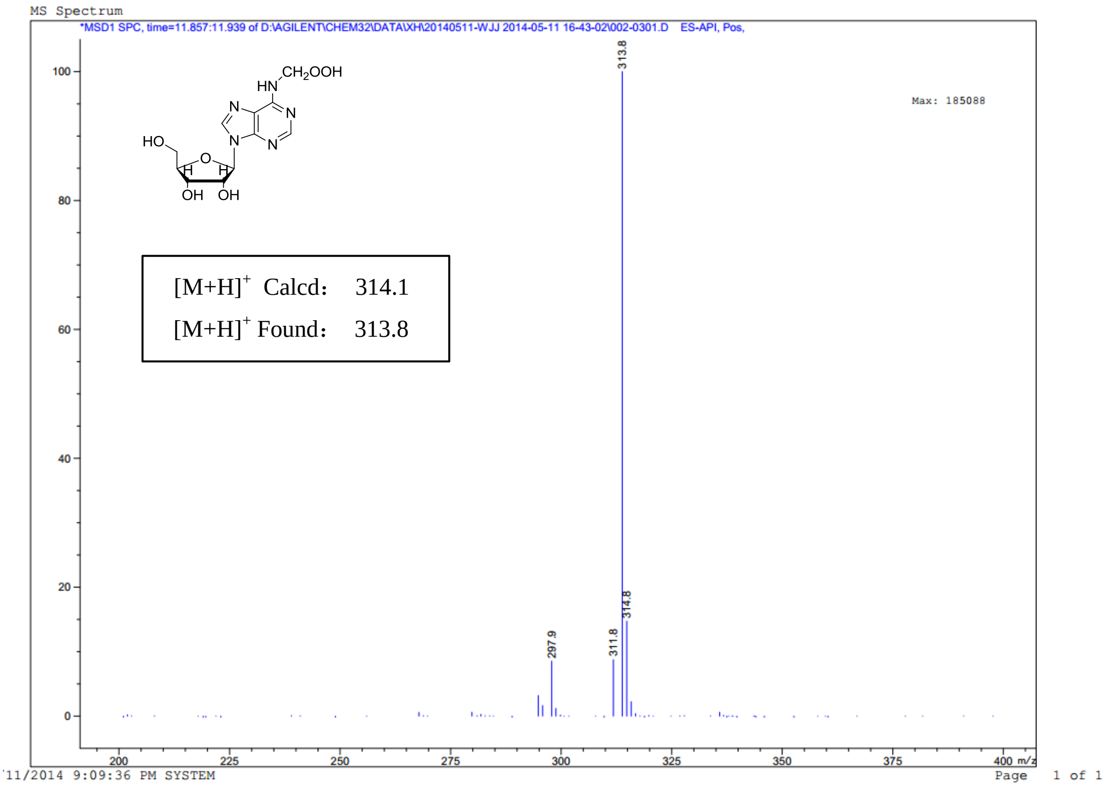

Figure S1. The LC-MS spectrogram of the reaction mixture at different retention time. The m/z at 297.8, 295.8 and 313.8 correspond to A (**a**), hm^{6}A (**b**), f^{6}A (**c**) and oxm^{6}A (**d**). The m/z 311.8 may correspond to the ca^{6}A

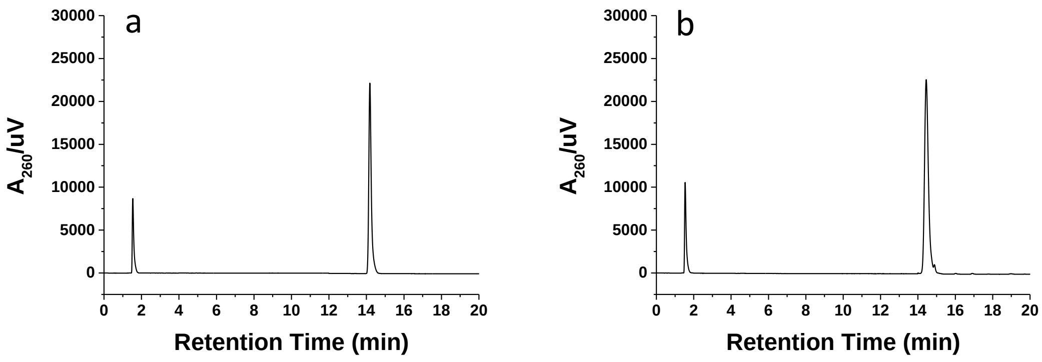

Figure S2. HPLC chromatograph of 2mM m^{6}A incubated with 200mM H_2O_2 at 37°C for 0h (**a**) and 24h (**b**).

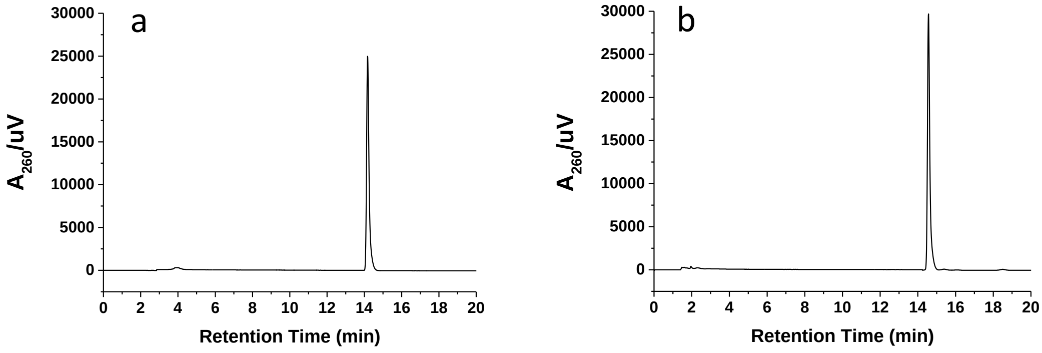

Figure S3. HPLC chromatograph of 2mM m^{6}A incubated with 1M NH_4HCO_3 at 37°C for 0h (**a**) and 24h (**b**).

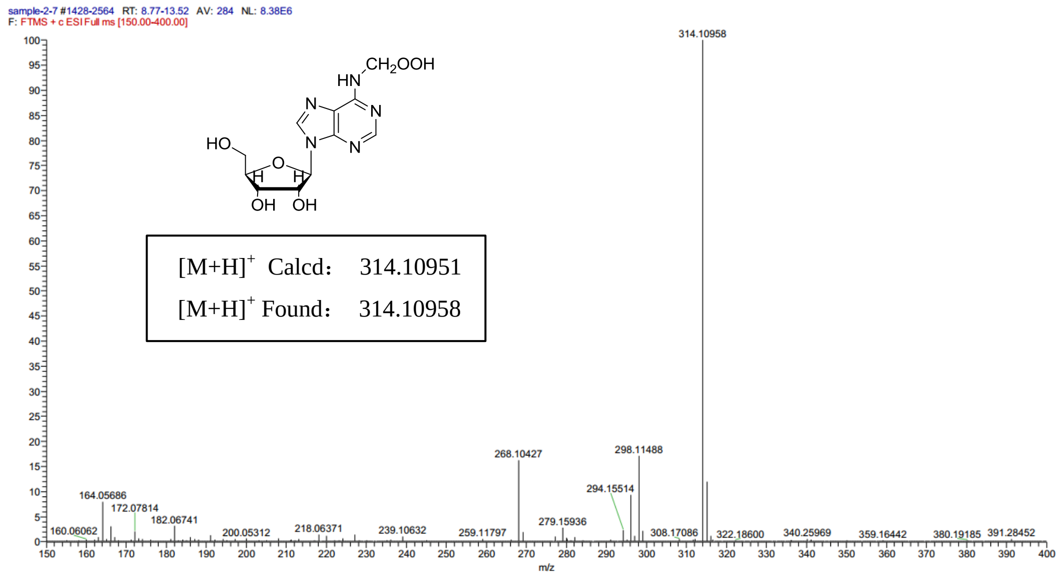

Figure S4. High-resolution mass spectrum of oxm^{6}A isolated by HPLC in positive mode.

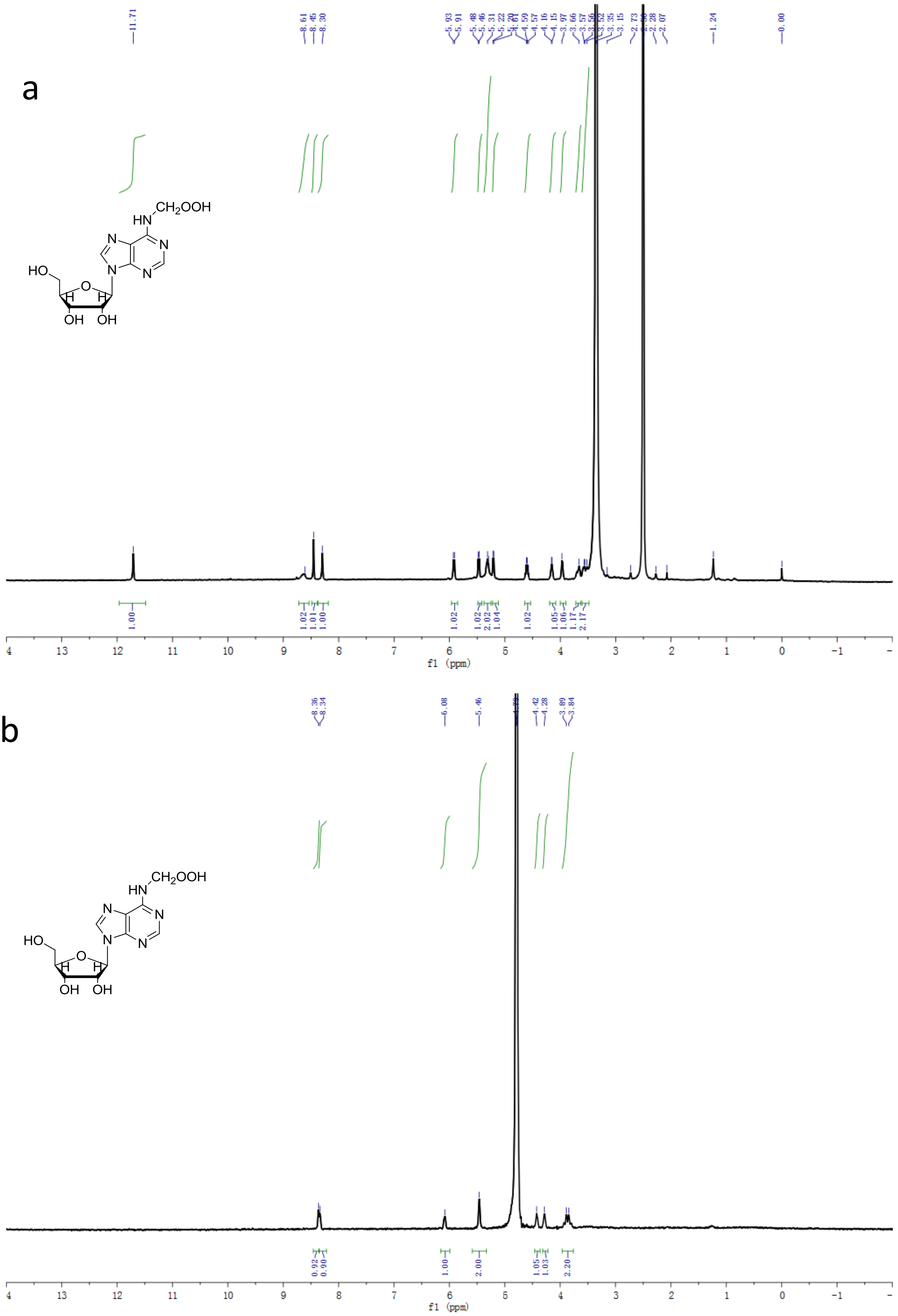
a
b
HN
CH2OOH
HO
OH OH
f1 (ppm)

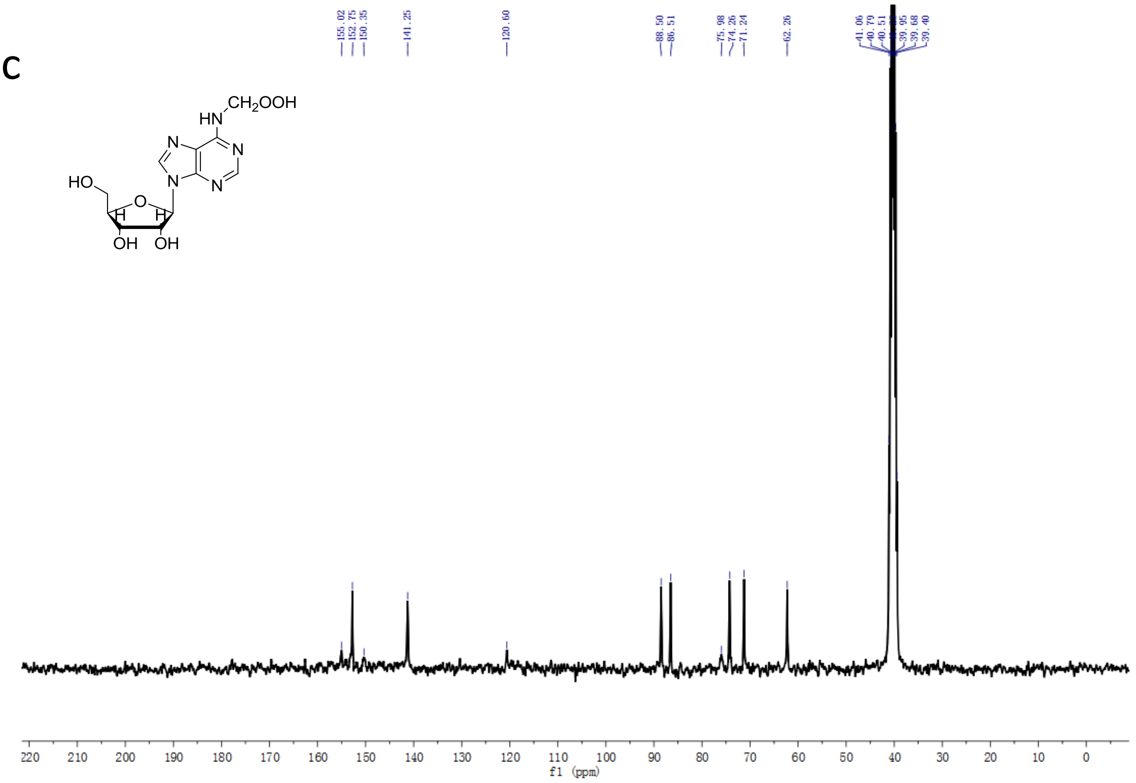

Figure S5. ^{1}H NMR spectra of oxm^{6}A in DMSO (a), D_2O (b) and ^{13}C NMR spectra in DMSO (c).

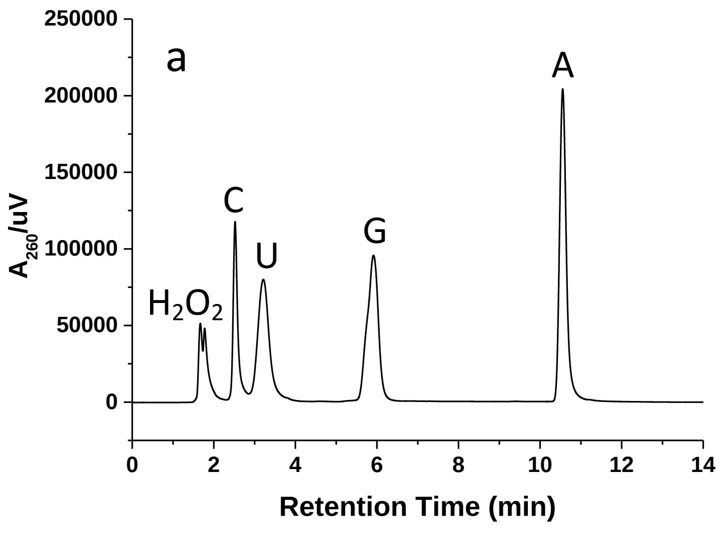

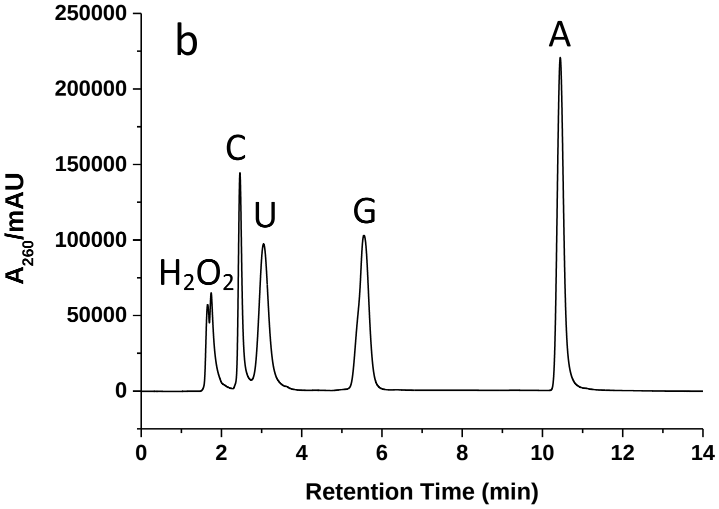

Figure S6. 2mM of A, U, C, G were treated with 200mM H_2O_2 and 1M NH_4HCO_3 for 1h (a: 0h, b: 1h), after reaction no new peak occurred, indicating the reagents have no side reaction with the four nucleosides under this conditions.

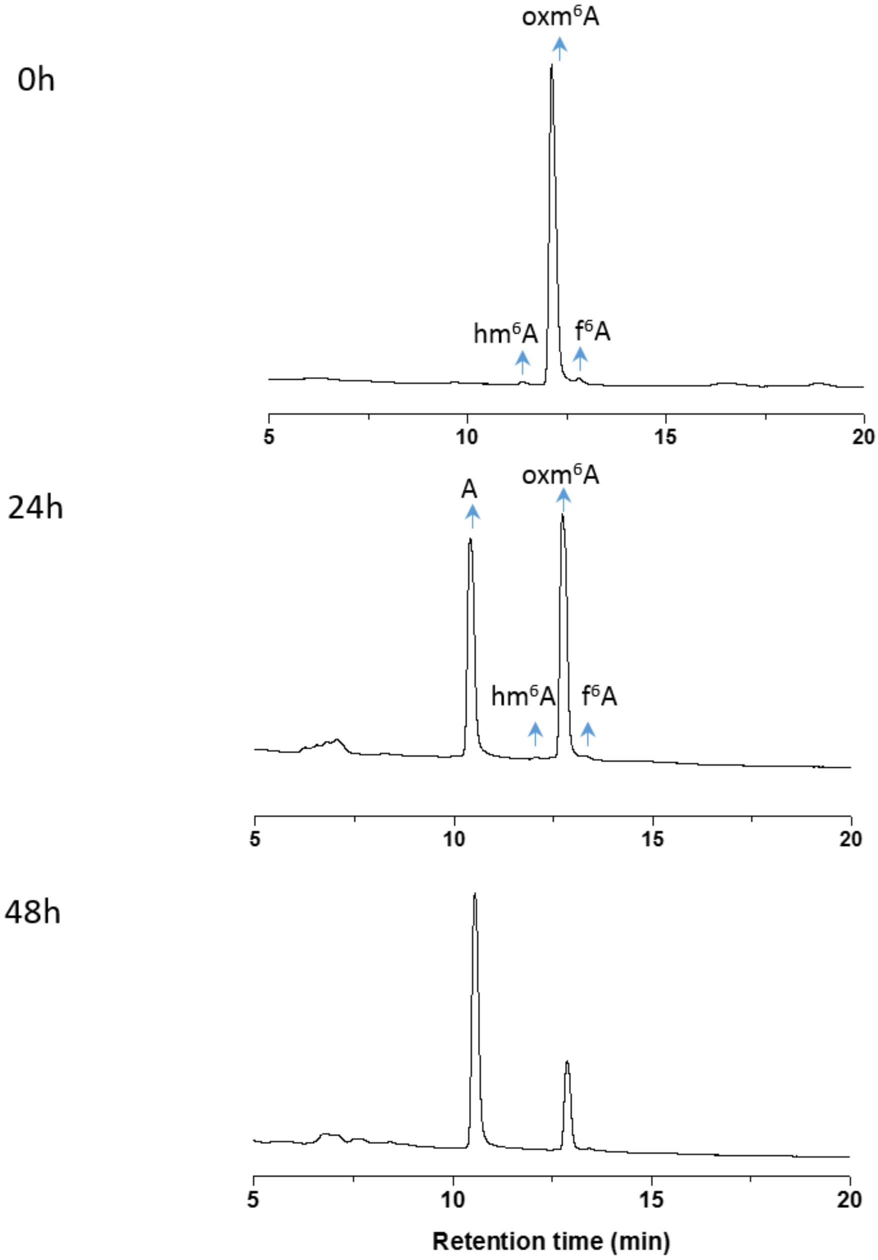

Figure S7. The decomposition of oxm^{6}A in the reaction condition monitored by HPLC chromatograms (260nm).

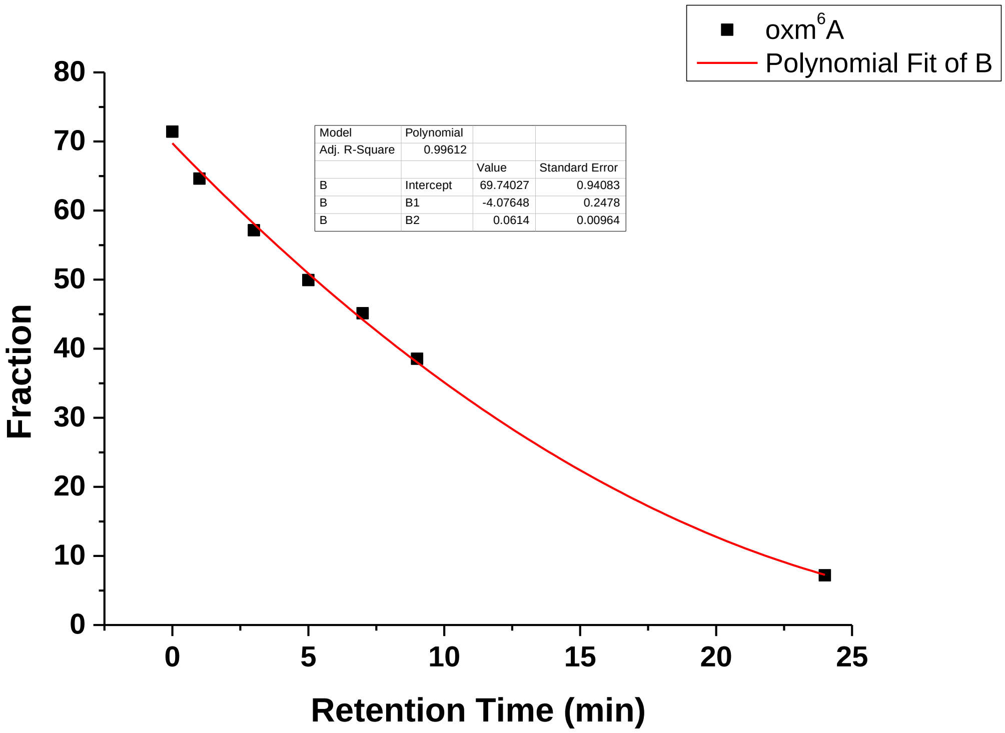

Figure S8. Time dependence of consumption of oxm[6]A in HEPES buffer (50mM, pH 7.4) at 37°C. Half–Life for decomposition of oxm[6]A was about 8.5h.

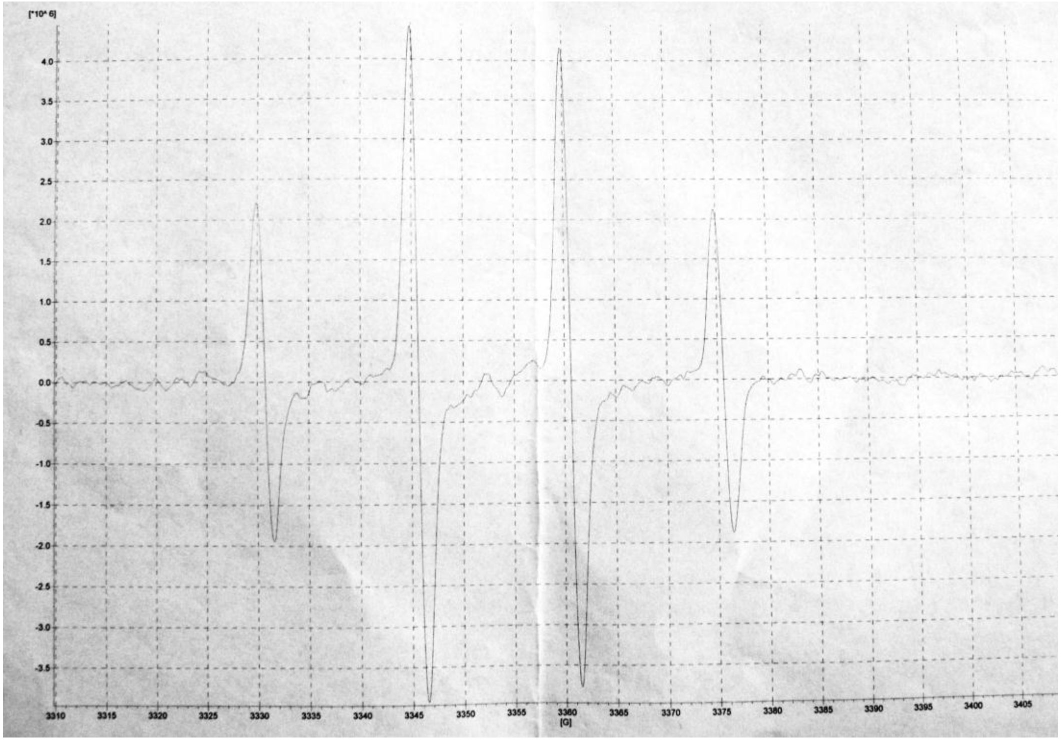

Figure S9. EPR spectra of H_2O_2 (200mM) /NH_4HCO_3(1M) system after incubated in 37°C for 15min.

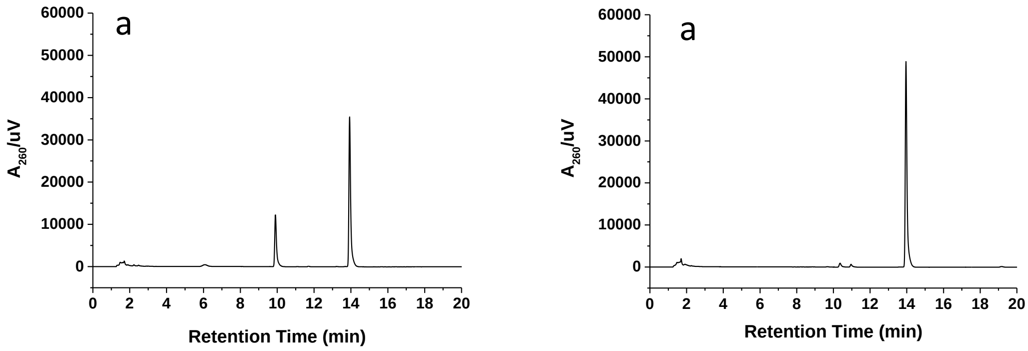

Figure S10. HPLC chromatograph of 2mM m^{6}A incubated with 200Mm H2O2 and 1M NH_4HCO_3 at 37°C in the absence (**a**) and presence (b) of DMSO (400mM)

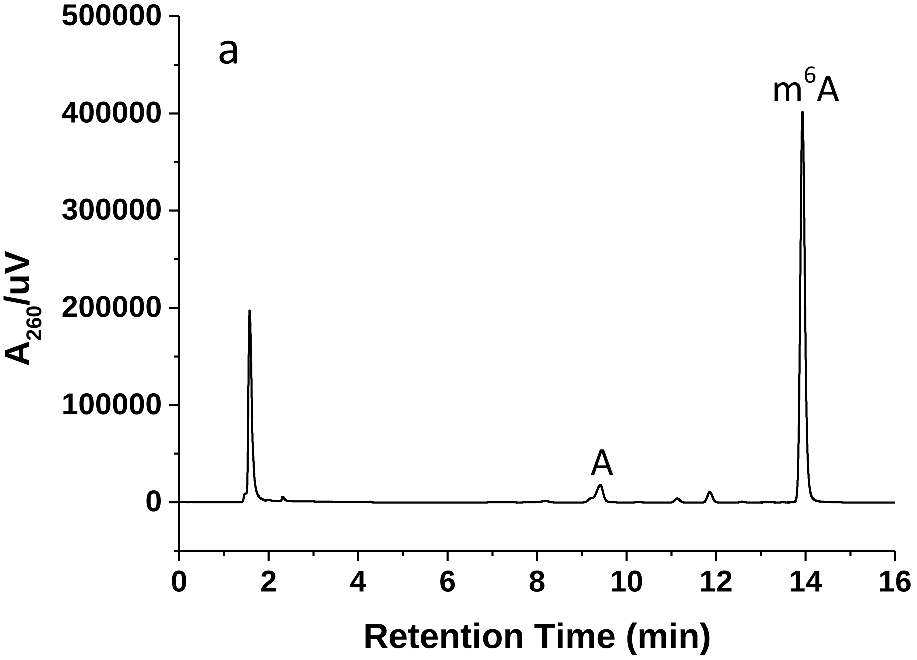

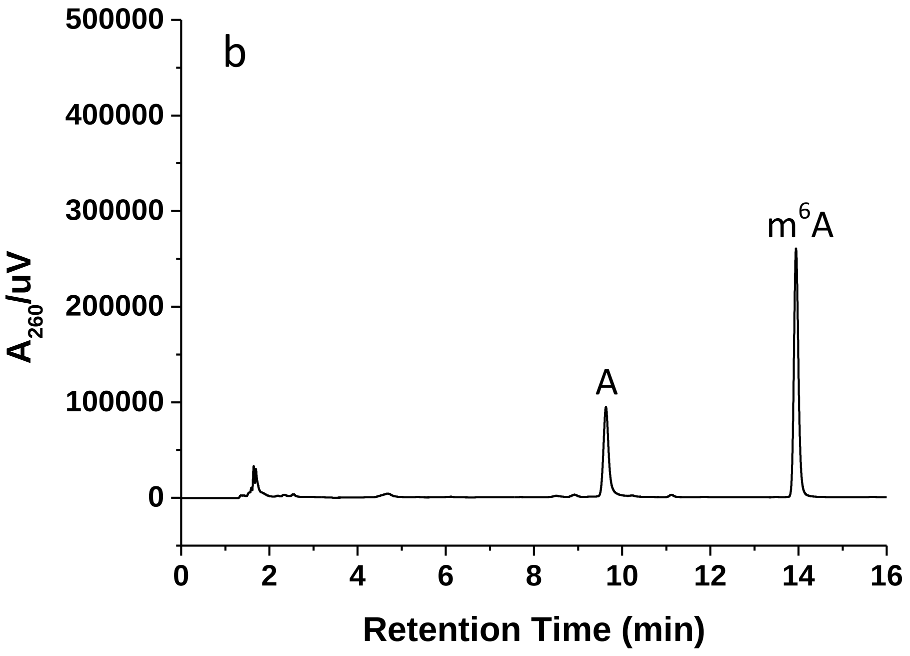

Figure S11. HPLC traces of the reaction of m^6A with NH_4HCO_3 (1M) and H_2O_2 (400mM) in the absence (a) and in the presence (b) of $(NH_4)_2Fe(SO_4)_2$ (4mM). The reaction with m^6A was carried out in 50mM HEPES buffer (pH 7.4; 37°C).

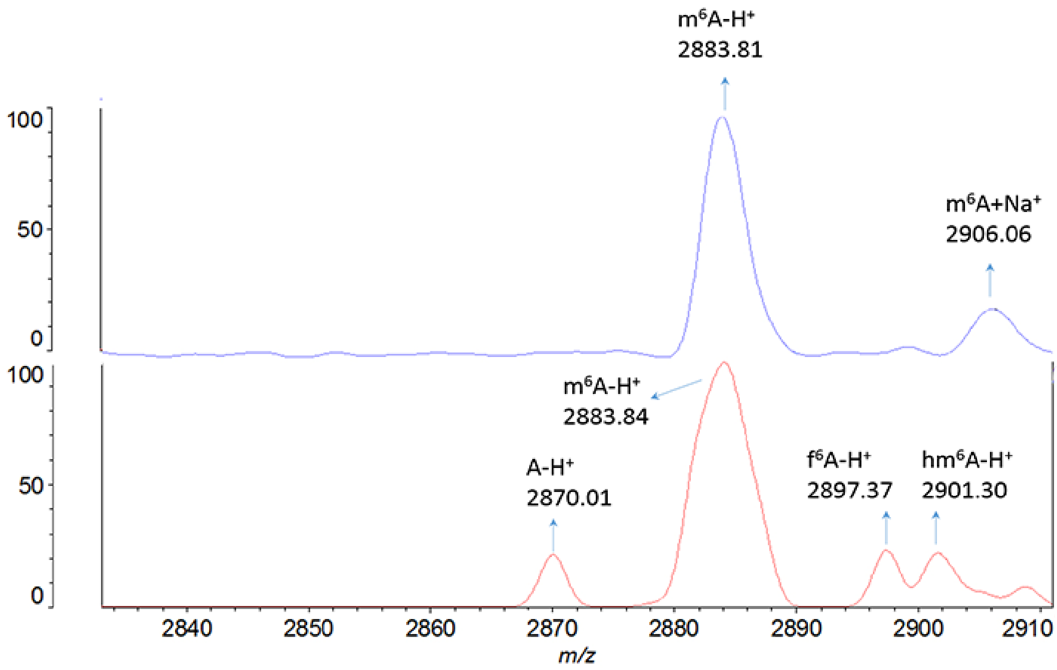

Figure S12. MALDI-TOF analysis of m^6A-RNA after reacting with H_2O_2/bicarbonate. After treating with 10mM H_2O_2 and 100mM NH_4HCO_3 for 48h, three new peak occurred, the m^6A-14Da correspond to the demethlate adenosine, the +14, and +17Da peak may represcent the formation of f^6A and hm^6A.

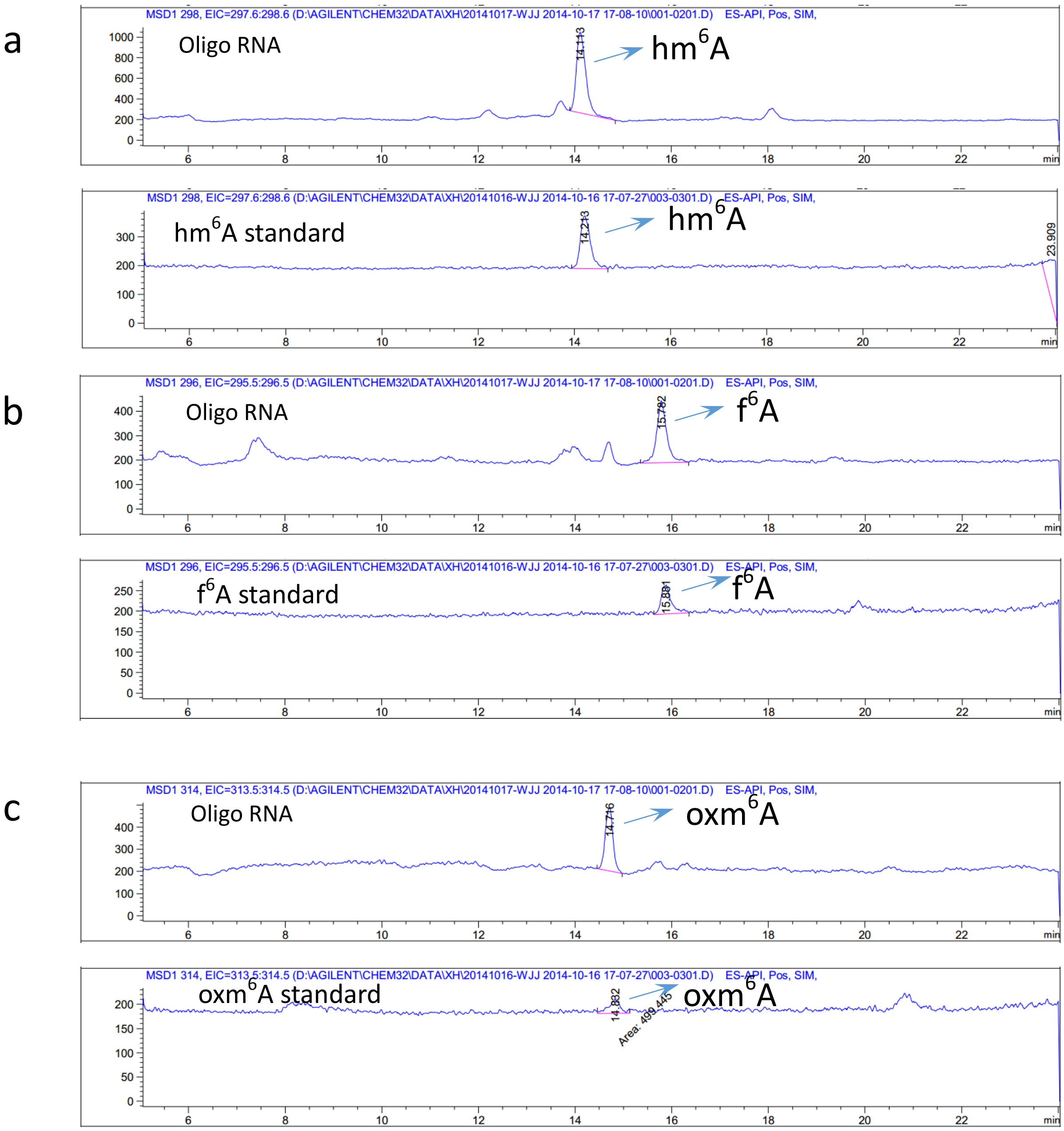

Figure S13. LC-MS analysis of the digested nucleosides in oligo RNA. After the reaction, we successfully find the presence of hm^{6}A (**a**), f^{6}A (**b**) and oxm^{6}A (**c**) in oligo RNA. The mass spectrometry detection was performed under single ion monitoring mode.

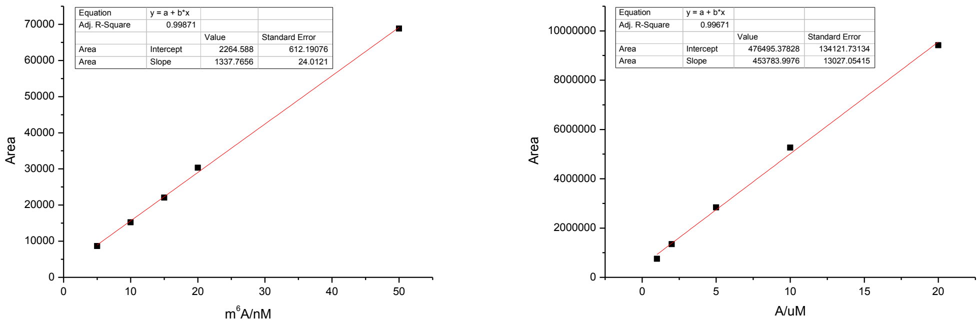

Figure S14. Quantification standard curve of m^{6}A and A standards in LC-MS.

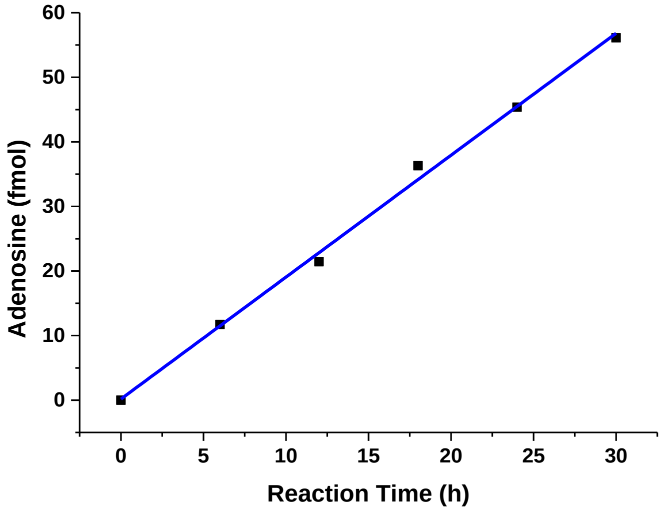

Figure S15. Time course of m^{6}A level in oligo RNA oxidized by H_2O_2/bicarbonate. Conditions: 2ug oligo RNA containing m^{6}A was incubated with 100 µM H_2O_2 and 300 µM NH_4HCO_3 at 37°C for 30h. After digestion, the mixture was analysed by LC-MS.

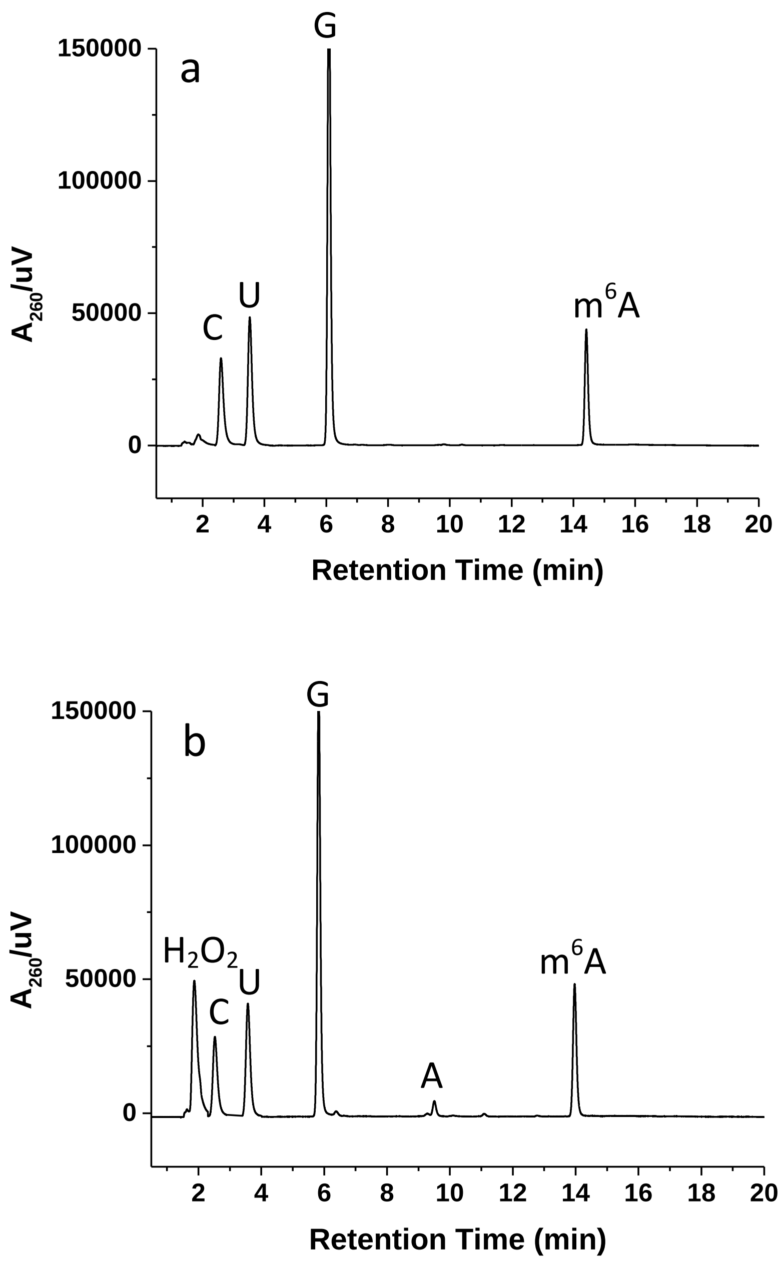

Figure S16. HPLC traces of digested nucleosides in single-stranded RNA, 5'-CUGGm^{6}ACUGG-3' without oxidation (a) and with H_2O_2 (50mM), Fe^{2+} (40 μM) and NH_4HCO_3 (50mM) treatment (b) in 50mM HEPES buffer at 37°C. After 1h, the peak of adenosine was observed.

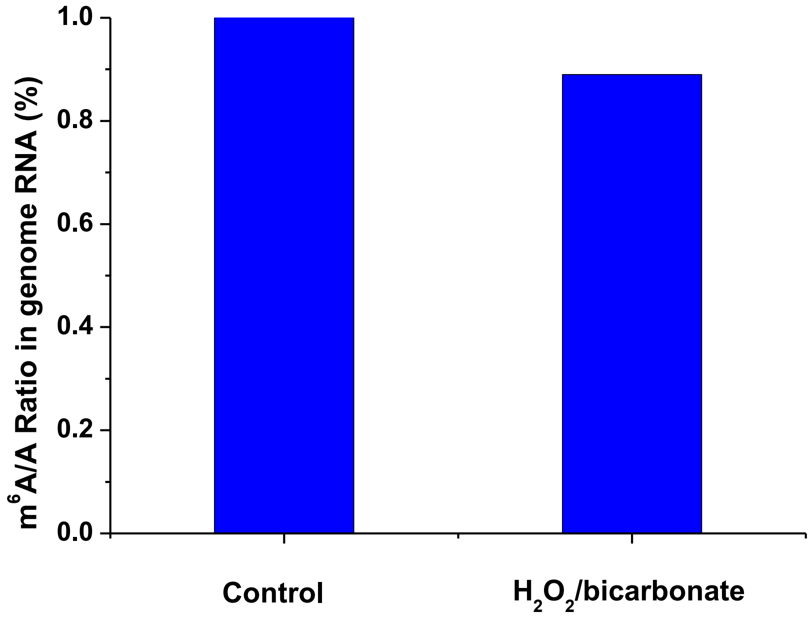

Figure S17. Quantification of m^6A/A ratio in Hela genome RNA by LC-MS. A decrease in m^6A/A ratio was observed in genome RNA with treatment of 50 μM H_2O_2 and 1mM NH_4HCO_3 at 37°C for 12h.

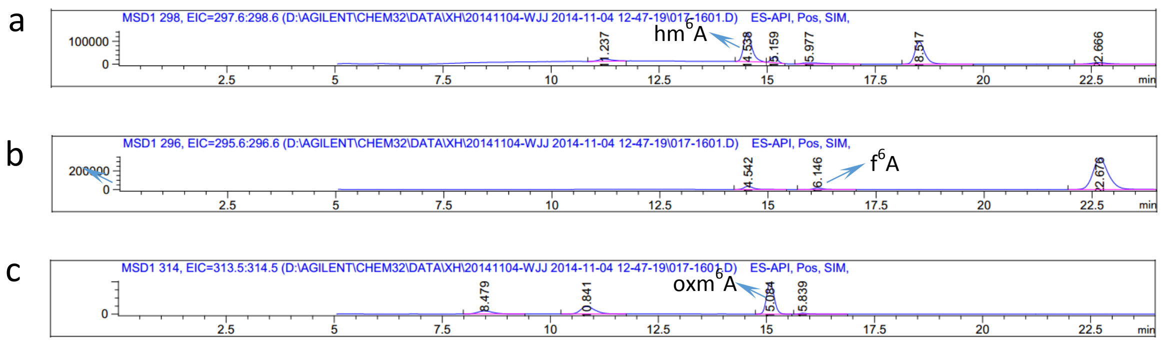

Figure S18. LC-MS analysis of the m^6A treated with Fenton-type reagent (1mM m^6A, 200mM H_2O_2, 40uM $(NH_4)_2Fe(SO_4)_2$) in oligo RNA at 37°C for 5h. After the reaction, we successfully find the presence of hm^6A (**a**), f^6A (**b**) and oxm^6A (**c**) in oligo RNA. The mass spectrometry detection was performed under single ion monitoring mode.

Table S1. Optimized operating conditions for ICP-OES.

RF generator power (W)	1150
Frequency of RF generator (MHz)	27.12
Coolant gas flow rate (L min^{-1})	14
Auxiliary gas (L min^{-1})	0.5
Plasma gas (L min^{-1})	0.6
Observation height (mm)	15
Max integration times (sec)	20
Analytical wavelength (nm)	Cu 324.754<br>Fe 259.940

Supplementary References:

1 B. Balagam; D. E. Richardson. *Inorg. Chem.* 2008, **45**, 1173-1178.

2 G. Ah-kow, F. Terrier, M. J. Pouet, M. P. Simonnin. *J. Org. Chem*. 1980, **45**, 4399-4404.

3 Y, Fu, G. Jia, X. Pang, R. N. Wang, X. Wang, C. J. Li, S. Smemo. Q. Dai, K. A. Bailey, M. A. Nobrega, K. L. Han, Q. Cui, C. He. *Nature Commun.* 2013, **4**, 1798-1806.
